# Cryo-EM structure of human Wntless in complex with Wnt3a

**DOI:** 10.1038/s41467-021-24731-3

**Published:** 2021-07-27

**Authors:** Qing Zhong, Yanyu Zhao, Fangfei Ye, Zaiyu Xiao, Gaoxingyu Huang, Meng Xu, Yuanyuan Zhang, Xiechao Zhan, Ke Sun, Zhizhi Wang, Shanshan Cheng, Shan Feng, Xiuxiu Zhao, Jizhong Zhang, Peilong Lu, Wenqing Xu, Qiang Zhou, Dan Ma

**Affiliations:** 1 Fudan University, Shanghai, China; 2 Key Laboratory of Structural Biology of Zhejiang Province, School of Life Sciences, Westlake University, Hangzhou, Zhejiang China; 3 Westlake Laboratory of Life Sciences and Biomedicine, Hangzhou, Zhejiang China; 4 Institute of Biology, Westlake Institute for Advanced Study, Hangzhou, Zhejiang China; 5 School of Life Science and Technology, ShanghaiTech University, Shanghai, China; 6 Mass Spectrometry Core Facility, The Biomedical Research Core Facility, Center for Research Equipment and Facilities, Westlake University, Hangzhou, Zhejiang China

**Keywords:** Cryoelectron microscopy, Cell signalling

## Abstract

Wntless (WLS), an evolutionarily conserved multi-pass transmembrane protein, is essential for secretion of Wnt proteins. Wnt-triggered signaling pathways control many crucial life events, whereas aberrant Wnt signaling is tightly associated with many human diseases including cancers. Here, we report the cryo-EM structure of human WLS in complex with Wnt3a, the most widely studied Wnt, at 2.2 Å resolution. The transmembrane domain of WLS bears a GPCR fold, with a conserved core cavity and a lateral opening. Wnt3a interacts with WLS at multiple interfaces, with the lipid moiety on Wnt3a traversing a hydrophobic tunnel of WLS transmembrane domain and inserting into membrane. A β-hairpin of Wnt3a containing the conserved palmitoleoylation site interacts with WLS extensively, which is crucial for WLS-mediated Wnt secretion. The flexibility of the Wnt3a loop/hairpin regions involved in the multiple binding sites indicates induced fit might happen when Wnts are bound to different binding partners. Our findings provide important insights into the molecular mechanism of Wnt palmitoleoylation, secretion and signaling.

## Abstract (web summary)

The transmembrane protein Wntless (WLS) is essential for the secretion of Wnt proteins, which are signaling molecules of the Wnt signaling pathways. Here, the authors present the 2.2 Å cryo-EM structure of human WLS in complex with Wnt3a and discuss mechanistic implications for the palmitoleoylation of Wnt3a that is required for WLS mediated Wnt secretion.

## Introduction

Wnt signaling is highly conserved among metazoans^1,2^, and controls many critical events during early embryonic development, later growth, maintenance of adult tissue homeostasis, as well as self-renewal of stem cells^3,4^. Working together with other signaling pathways, Wnt signaling drives cell proliferation and differentiation in an appropriate manner, which is crucial for all the multicellular organisms. Mutations in Wnt signaling pathway genes that cause aberrant signaling activities are tightly associated with many human diseases including some aggressive metastatic cancers^5,6^.

Wnt proteins comprise a family of secreted proteins^7,8^. They bind to highly conserved cell surface receptors^9–12^ and trigger signaling cascades in target cells in either paracrine or autocrine manner^13^. Wnt proteins harbor a palmitoleate (PAM) modification in most circumstances, which is catalyzed by the *O*-acyltransferase Porcupine during Wnt maturation in ER^14–17^. The subsequent secretion of Wnt proteins into extracellular space relies on an evolutionarily conserved multi-pass transmembrane protein Wntless (WLS)^18–20^.

WLS has been found to directly interact with Wnts throughout the secretory route initiated from ER, then Golgi, secretory vesicles and finally cell membrane^18,19,21,22^. After release of Wnts, WLS is recycled from cell membrane to ER by a retromer complex^23–25^. WLS is essential for Wnt secretion and downstream signaling, however, due to lack of structural information, the molecular mechanism of Wnt secretion remains poorly understood. It has also been shown that WLS is upregulated in some cancers and promote caner development, indicating WLS is a potential drug target for the treatment of Wnt-driven cancers^26–28^. Three-dimensional (3D) structures of WLS and WLS-Wnt complexes are in need for understanding the mechanism for Wnt-secretion, as well as the discovery of new cancer therapeutics targeting Wnt signaling.

Here, we report a cryo-EM structure of wild type (WT) human WLS in complex with Wnt3a at 2.2 Å resolution. Wnt3a bound to WLS exhibits structural differences compared to the structure of its close homolog Wnt3 in the complex with cysteine-rich domain of its receptor Frizzled (FZD-CRD). Notably, during preparation of this manuscript, an EM structure of human WLS-Wnt8a complex was reported^29^, with a similar overall structure to that of the WLS-Wnt3a complex. A comparison of the two WLS-Wnt structures provides structural basis for understanding the molecular mechanism of WLS-mediated Wnt secretion, Wnt signaling, and development of drugs for treatment of Wnt-driven cancers.

## Results

### Overall structure of the human WLS-Wnt3a complex

We co-expressed full-length human WLS and Wnt3a in HEK293S GnTI^−^ cells co-infected with viruses generated from pEG BacMam vector^30^. In order to trap WLS-Wnt3a complex within cells, we treated the transfected cells with V-ATPase inhibitor bafilomycin A1 to suppress Wnt secretion^22,31^. We purified the WLS-Wnt3a complex by affinity chromatography followed by size exclusion chromatography (SEC). The homogeneity of protein samples was checked through negative staining for initial screening before cryo-EM sample preparation and data acquisition.

**Fig. 1:**
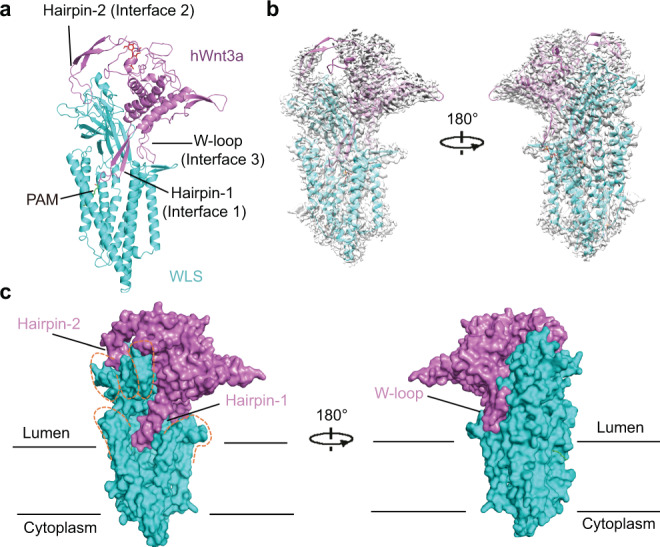
Cryo-EM structure of human WLS in complex with Wnt3a. **a** Overall structure of WLS-Wnt3a complex. Structure is shown in cartoon with WLS colored in cyan and Wnt3a colored in violet. The sugar moiety on Wnt3a is shown as red sticks, and PAM modified on Wnt3a is shown as green sticks. Key structural elements of Wnt3a at interfaces are indicated. **b** Fit of WLS-Wnt3a complex model with the 2.2 Å cryo-EM map. Wnt3a is colored in violet and WLS is colored in cyan. The map is generated from the 2.2 Å map in Chimera^65^ at contour level of 0.2 with dust hidden. **c** Complex structure is shown in surface. Model is colored in same pattern as in Fig. 1b. The contour of hand shape of WLS is shown in orange dash lines. All structural figures are generated with PyMOL^66^.

After data processing, we obtained a 2.2 Å resolution cryo-EM map (Supplementary Figs. 1, 2). We used a human Wnt3 structure model (hWnt3, PDB code: 6AHY) and a de novo predicted model of WLS (generated from trRosetta^32^ and tFold^33^) to guide the model building. The built model comprises 496 residues of 541-residue WLS and 334 residues of Wnt3a after cleavage of signal peptide (Fig. 1a and Supplementary Table 1). In our model, WLS contains 8 helices and 8 β-strands, whereas Wnt3a contains 7 helices and 6 β-strands. The model of WLS-Wnt3a complex fits well with the cryo-EM map (Fig. 1b and Supplementary Figs. 3, 4). The missing densities for flexible regions in the 2.2 Å map (Fig. 1b, at contour level of 0.2) can be seen in an additional 3.2 Å map (Supplementary Fig. 3a, at contour level of 0.02).

In the overall structure, Wnt3a perches on the luminal side of WLS with several β-hairpin and loop regions (Hairpin-1, Hairpin-2 and W-loop) interacting with WLS extensively and generate three major interfaces (Fig. 1). The complex structure resembles two hands grabbing with each other. WLS looks like a grasping right hand that seizes one finger (Hairpin-1) of Wnt3a bearing the conserved palmitoleoylated Ser209, which is reported to be essential for Wnt secretion and interaction with Frizzled receptors on cell membrane^34^. Wnt3a pinches WLS with another two fingers (Hairpin-2 and W-loop) at the luminal region of WLS (Fig. 1c). Wnt3a is palmitoleoylated with density for PAM clearly resolved (Supplementary Fig. 4), and the modification was confirmed by mass spectrometry (Supplementary Fig. 5).

### Structural features of WLS

**Fig. 2:**
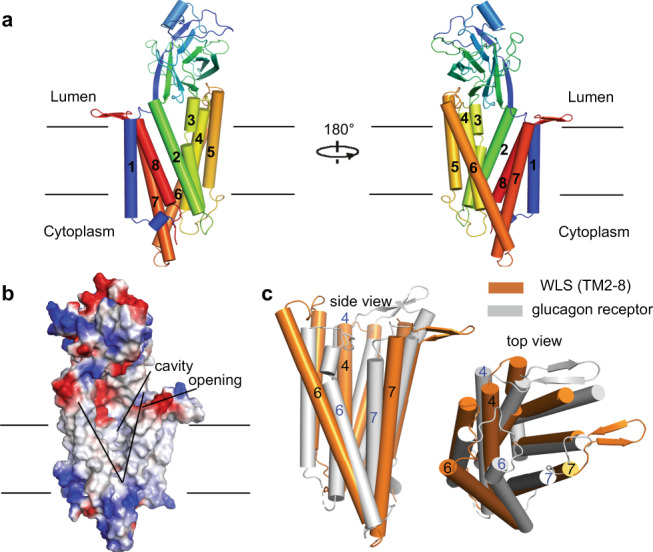
Structural features of WLS. **a** Overall structure of WLS. WLS is shown in cartoon with cylindrical helices and rainbow colored. Helix numbers are labeled. **b** Electrostatic analysis result. The cavity and lateral opening are indicated. **c** Superimposition of TM2-8 of WLS and TM region of glucagon receptor (PDB code: 5XF1). Both side view and top views are shown.

In our model, WLS contains an 8-spanning transmembrane (TM) domain, with both of its N- and C-termini on the cytoplasmic side, and the luminal soluble domain lies between the first and second transmembrane helix (Fig. 2a).

Electrostatic analysis indicates the TM domain of WLS is very hydrophobic, whereas the luminal domain and the cytoplasmic side are highly charged (Fig. 2b). Intriguingly, we found in the TM domain of WLS there is a large hydrophobic cavity and a lateral opening between TM6 and TM7 that exposes the cavity to membrane environment (Fig. 2a, b). This cavity accommodates the extruding Hairpin-1 of Wnt3a (Fig. 1c), and one POPC molecule, clearly identified in the EM map (Supplementary Fig. 6a).

By searching the structure of WLS on the Dali server^35^, we found that the top hits are some well-known representatives of GPCR family, like glucagon receptor^36^, smoothened^37^ and Frizzled-4^38^. We superimposed WLS TM2-8 and TM region of glucagon receptor (PDB code: 5XF1) (Fig. 2c). TM4, TM6 and TM7 in WLS have the most significant conformational differences compared to those in glucagon receptor: TM4 moves towards the core of TM domain, reducing the volume of the internal cavity; TM6 and TM7 move away from each other, generating the lateral opening of WLS. These results suggest TM2-8 of WLS adopts a GPCR fold, though the sequence identity is relatively low (around 10% or even lower).

The luminal domain of WLS (residue range 36–227) is arranged into a β-sandwich fold and contains two disulfide bonds that stabilize the structure. The luminal domain shares structural similarity to the monomeric form of a lipid binding protein Seipin^39^ (PDB code: 6DS5, Supplementary Fig. 6b), which is involved in lipid droplet biogenesis.

### WLS/Wnt3a interaction interfaces

**Fig. 3:**
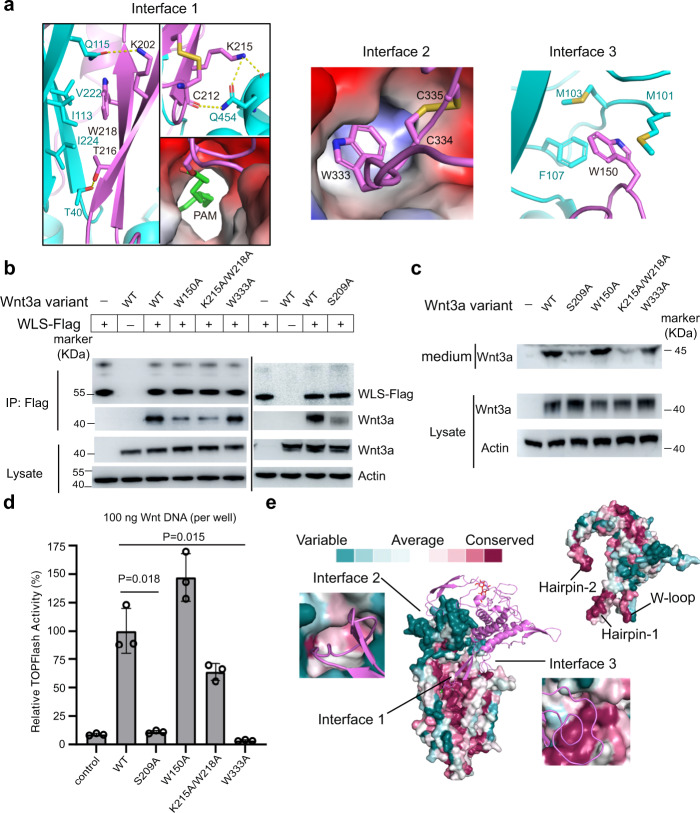
Interfaces between WLS and Wnt3a. **a** Three major interfaces between WLS and Wnt3a and the interaction details. Residues are indicated as one-letter codes here and in all other figures with specific residues highlighted. **b** Interaction validation between WLS and different Wnt3a variants through co-IP. In total 2.5 µg /well (six-well plate) of plasmids were co-transfected into Hela cells with Wnt3a: WLS DNA ratio at 1:1. Experiments were independently performed for three times with similar results. **c** Secretion of Wnt variants, detected by western blotting. Experiments were independently performed for three times with similar results. **d** Signaling activity for different Wnt3a variants. Activity was measured by TOPFlash luciferase reporter assay. Normalized activity for WT Wnt3a is taken as 100%, and activity of Wnt3a mutants is shown as percentage activity compared to WT Wnt3a. 100 ng of Wnt3a DNA per well (24-well plate) was used during transfection. All histograms were generated from *n* = 3 independent measurements by GraphPad Prism 9. Statistical analysis was performed by two-sided test; mean ± S.D. **e** Conservation surface of Wnt3a and WLS. The highly conserved regions involved in binding at interfaces are indicated.

Wnt3a interacts with WLS mainly at three major interfaces mediated by Hairpin-1 at Interface 1, Hairpin-2 at Interface 2 and W-loop at Interface 3, respectively (Fig. 1a). The density in cryo-EM map allowed us to identify interaction details (Figs. 3a and Supplementary Fig. 3, 4).

At Interface 1: Lys202 of Wnt3a forms a hydrogen bond with Gln115 in luminal domain of WLS; Cys212 and Lys215 in Wnt3a interacts with Gln454 in WLS through hydrogen bonds; Thr216 of Wnt3a forms a hydrogen bond with Thr40 of WLS; Trp218 of Wnt3a interacts with Ile113, Val222 and Ile224 in the luminal domain of WLS through hydrophobic interaction; the PAM group attached to Wnt3a Ser209 inserts into a hydrophobic tunnel formed between TM4 and TM5 of WLS (Fig. 3a and Supplementary Fig. 7a). At Interface 2, Wnt3a Hairpin-2, containing Trp333 and stabilized by the disulfide bond formed between Cys334 and Cys335, extensively interacts with residues in a hydrophobic pocket at the distal region of WLS luminal domain (Fig. 3a and Supplementary Fig. 7b). In addition, His332 on Wnt3a Hairpin-2 forms a hydrogen bond with Glu205 of WLS. At Interface 3, Trp150 on Wnt3a W-loop forms hydrophobic interactions with Met101, Met103, and Phe107 in the loop between β-strand 2 (β2) and β-strand 3 (β3) of WLS luminal domain (Fig. 3a). At the three major interfaces, we also found some intermolecular hydrogen bonds between Wnt3a Hairpin-2/ W-loop and WLS, involving some mainchain polar atoms.

We introduced several interface-mutations in Wnt3a and examined WLS interaction, Wnt secretion and downstream Wnt signaling. Co-IP results suggest that mutation of W150A at W-loop, and S209A, K215A/W218A at Hairpin-1 significantly impaired interaction with WLS, whereas W333A mutation at Hairpin-2 has little influence on WLS-Wnt3a binding (Fig. 3b). Palmitoleoylation on Ser209 of Wnt3a is thought to be required for WLS-Wnt3a association^22,40^. However, we found the binding between Wnt3a S209A mutant and WLS is dependent on the expression level of Wnt3a mutant: the more S209A mutant plasmid was transfected, the more mutant protein could be co-immunoprecipitated with WLS (Supplementary Fig. 7c, d). Co-expression and purification results demonstrate Wnt3a S209A mutant can form stable complex with WLS on gel filtration (Supplementary Fig. 7e). We also detected Wnt secretion level and Wnt signaling activity for different Wnt variants (Fig. 3c, d and Supplementary Fig. 7f). Results suggest that both S209A and K215A/W218A mutations at Hairpin-1 heavily decreased Wnt3a that are secreted into medium, whereas W333A mutation at Hairpin-2 had little influence on Wnt secretion (Fig. 3c). In a TOPFlash assay, Wnt signaling activity was nearly abolished for S209A mutant and W333A mutant, whereas K215A/W218A mutant kept substantial signaling activity (Fig. 3d). Intriguingly, although W150A mutation at W-loop weakened the binding between Wnt3a and WLS, there was little influence on both Wnt secretion and Wnt signaling activity.

To further analyze the interactions between Wnt3a and WLS, we generated conservation surface of Wnt3a based on the alignment of all 19 human Wnt sequences, using the ConSurf server^41^ (Fig. 3e and Supplementary Fig. 8). Results show that the three regions of Wnt3a (Hairpin-1, Hairpin-2, and W-loop) that participate in the interactions with WLS are highly conserved among human Wnts. We also generated conservation surface for WLS based on the alignment of WLS sequences from various species (Fig. 3e and Supplementary Fig. 9), which indicates that all the regions of WLS at the interaction interfaces are conserved.

### Conformational differences between Wnt3a and Frizzled bound Wnt3

**Fig. 4:**
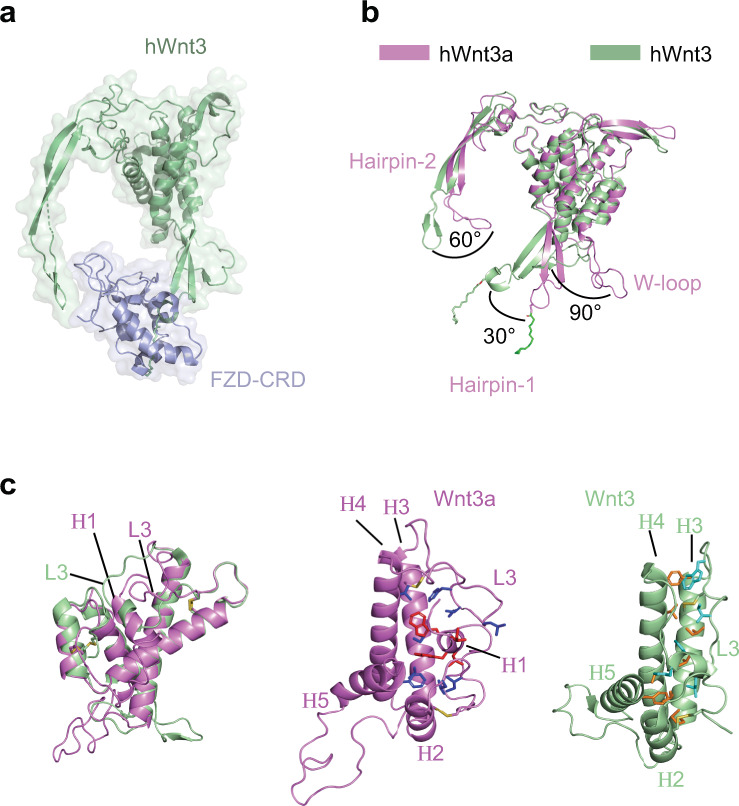
Comparison between WLS bound Wnt3a and FZD-CRD bound Wnt3. **a** Structure of hWnt3 in complex with FZD-CRD^42^. **b** Superimposition of hWnt3a and hWnt3. **c** Comparison of N-terminal regions of Wnt3a and Wnt3. In left panel, N-half regions are isolated from superimposition result of Wnt3a and Wnt3. In right panel: For Wnt3a, residues of H1 are colored in red and shown as sticks, and interacting residues are colored in blue. For Wnt3, residues of L3 are colored in cyan and shown as sticks, and interacting residues are colored in orange. Disulfide bonds are shown as sticks.

We compared the structures of WLS bound Wnt3a and its close homolog Wnt3 in complex with FZD-CRD^34,42^. Human Wnt3 (hWnt3, PDB code: 6AHY) clamps FZD-CRD via corresponding Hairpin-1/W-loop and Hairpin-2, and the PAM on Wnt3 directly interacts with FZD-CRD (Fig. 4a). Superimposition of structures of Wnt3a and Wnt3 reveals dramatic conformational differences in the loop and hairpin regions of Wnt that are involved in the binding with WLS. Compared to Wnt3a bound with WLS, Hairpin-1 of Wnt3 undergoes a 30° rotation, while Hairpin-2 and W-loop rotate by about 60° and 90°, respectively (Fig. 4b).

Significant conformational differences are also found in N-terminal region (Fig. 4c). Wnt3 in its crystal structure lacks the N-terminal region of residues 19–41, whereas in our model the N-terminal region of Wnt3a is well resolved in the EM map (Supplementary Fig. 4). We found Wnt3a residues 22–26 form a small 3_10_ helix (H1) and extensively interact with surrounding helices (H2 to H5) and loop3 (L3) between H3 and H4 of Wnt3a, mainly through hydrophobic interactions (Fig. 4c). In FZD-bound Wnt3 structure, in which more N-terminal residues are removed, L3 is translocated and occupies the original position of H1, with new hydrophobic interactions generated between L3 and other structural elements (Fig. 4c).

## Discussion

We have determined the cryo-EM structure of the human WLS-Wnt3a complex. In this structure, TM2-8 of WLS exhibits a GPCR-like fold (Fig. 2c), whereas TM1 may play a structural role and help stabilize the GPCR-like fold and the luminal domain between TM1 and TM2. Notably, we identified a lateral opening and a large cavity in the TM domain of WLS (Fig. 2b). It is possible that Hairpin-1 of Wnt3a enter through this opening to the core cavity, especially when modified with a PAM.

Compared to Wnt8a in complex with WLS^29^ (PDB code: 7KC4), Wnt3a bound to WLS has an extended N-terminal structure, while remaining region is similar (Supplementary Fig. 10a). A comparison of structures of WLS in the two complexes revealed few conformational differences, though the structures are solved in GDN detergent and nanodisc for WLS-Wnt3a and WLS-Wnt8a, respectively (Supplementary Fig. 10b). WLS-Wnt8a complex generally shares the same interacting mode with WLS-Wnt3a complex (Supplementary Fig. 10c). In WLS-Wnt8a structure, the interactions between WLS and Wnt8a at three major interfaces are also mediated by the three structural elements corresponding to Hairpin-1, Hairpin-2 and W-loop in our Wnt3a structure. Above results and conservation analysis (Fig. 3e and Supplementary Figs. 8, 9) suggest different Wnt proteins may interact with WLS in a similar way.

Compared to Wnt8a, Wnt3a/Wnt3 have longer N-terminal region (Supplementary Fig. 8), removal of which has been demonstrated not affect Wnt secretion but dramatically decrease the signaling activity^42,43^. In Wnt3a, N-terminal H1 seems to play a structural role and stabilize neighboring structures, especially the two disulfide bonds formed by Cys42/Cys56, which is absent in Wnt8a, and Cys77/Cys88 (Fig. 4c). Our structural findings are in consistence with previous studies which suggested the removal of Wnt3a N-terminal residues 19-26 may result in oxidized Wnt oligomers and minimized signaling activity by forming intermolecular disulfide bonds^43^. In crystal structure of Wnt3 with a truncated N-terminus, the structure seems to be stabilized in a different way, in which part of L3 of Wnt3 forms a helical structure occupying the position that H1 takes in the structure of Wnt3a (Fig. 4c). These conformational differences may be a result of the flexible nature of the loop regions. To further investigate the roles of N-terminal region, more systematic structural and functional studies need to be performed in the future.

Mutagenesis of Wnt3a resulted in complicated phenotype in functional assays, including interaction with WLS, secretion, and signaling (Fig. 3). K215/W218A mutations of Wnt3a at Interface 1 impaired interaction with WLS and reduced Wnt3a secretion, which is consistent with structural findings in this study. Despite poor binding and secretion, K215A/W218A mutant exhibited ~60% signaling activity of WT, likely due to the reduced amount of Wnt3a mutant is still sufficient to activate endogenous receptors. This result agrees with that the corresponding residues in Wnt3 are not involved in binding with FZD-CRD^34,42^ (Supplementary Fig. 11). The phenomenon that mutation of palmitoleoylation site Ser209 at Interface 1 and Trp 333 at Interface 2 of Wnt3a resulted in abolishment of signaling activity, is in consistence with previous findings that both PAM and the conserved Trp residues in other Wnts are directly involved in binding between Wnt and FZD-CRD^34,42^ (Supplementary Fig. 11). Mutation of Wnt3a Trp150, which is involved in binding with WLS but not FZD-CRD (Supplementary Fig. 11), substantially reduced binding of Wnt3a to WLS in co-IP experiments, while secretion and signaling remained minimally affected, indicating interactions at Interface 3 may not be necessary for Wnt secretion and signaling.

Conformational differences between Wnt3a in our structure and Frizzled bound Wnt3 (Fig. 4) indicates a structural rearrangement of the loop and hairpin regions in Wnt is required during Wnt transfer from WLS to FZD receptors at cell surface. These structural differences might reflect an induced fit mechanism when Wnt is bound to different binding partners. Wnt may be transferred directly from WLS to FZD or through facilitated diffusion mediated by cargo proteins after release from WLS^44–48^. As PAM modified Hairpin-1 and Haripin-2 of Wnt can interact with both WLS and FZD, these structural elements need to be released from WLS first and then bind to FZD, which may be facilitated by Wnt cargo proteins and/or co-receptors like LRP6. The exchanging of these shared interfaces may be coupled with large conformational changes.

The structure of WLS-Wnt3a complex we present here provides important insights into the mechanism of Wnt secretion and signaling. The regions of Wnt3a that are involved in the binding between WLS are highly conserved among all human Wnts, and interfaces on WLS are also conserved among species, suggesting WLS-mediated Wnt secretion probably share conserved mechanisms. The luminal domain of WLS provides multiple binding sites for Wnt3a, while the TM domain provides a core cavity which is ideal for accommodation of the tip of Hairpin-1 with or without PAM. Containing a GPCR fold and the existence of a conserved central cavity make WLS a potential drug target for the treatment of Wnt-driven cancers. Secretion of Wnts also involves some other important regulatory proteins^49,50^, for example those participating in vesicle trafficking. How Wnt-WLS complex interact with other regulators, and how Wnts are released from WLS and transferred to their receptors or other extracellular binding proteins remain unclear. More future studies are in need to answer these questions.

## Methods

### Protein expression and purification

cDNA of human WLS, Wnt3a and Porcupine were cloned into PEG BacMam vector^30^ (gifted from Dr. Eric Gouaux) and bearing C-terminal Flag tag, 10xhis tag and GFP-Flag tag, respectively. Primers for cloning are shown in Supplementary Table 2. BacMam viruses were generated through standard methods and batches of P4 were used for transfection of HEK293S GnTI^−^ cells. Plasmid containing Porcupine was transiently transfected into HEK293S GnTI^−^ cells at density of 2.0 × 10^6^/ml. Then volume ratio of 1: 4 for viruses of WLS and Wnt3a were supplemented. After 8–12 h of post-transfection, 10 mM of sodium butyrate and 100 nM of bafilomycin A1 was supplemented and culture temperature was lowered to 30 °C. After another 48 h, cells were harvested and resuspended with lysis buffer containing 25 mM Tris, pH 8.0, 150 mM NaCl and protease inhibitors including Aprotinin, Pepstatin, Leupeptin and PMSF. Cells were disrupted by sonication followed by adding detergent GDN at final concentration (w/v) of 1% for membrane protein extraction at 4 °C for 2 h. After centrifugation at 100,000 g for 1 h, we collected the supernatant for further purification by anti-Flag agarose and then nickel resin before run gel filtration in buffer containing 25 mM Tris, pH 8.0 and 150 mM NaCl and 0.03% GDN. All DNA and protein sequences were analyzed with SnapGene.

### Cryo-EM sample preparation and data collection

The purified WLS-Wnt3a complex at concentration of 0.19 mg/ml was used for cryo-EM sample preparation. Holey carbon grids (Quantifoil Au R1.2/1.3) were coated with a thin layer of graphene oxide^51^. Briefly, graphene oxide (Sigma-Aldrich, 763705, 2 mg/ml) was diluted to 0.02 mg/ml, and water-bath sonicated for 1 min before centrifuged to remove any possible aggregates. Then, 1 μl of the above supernatant was loaded onto each glow-discharged Quantifoil grid for air-dry. In total, 3 μl of protein sample was applied onto each grid followed by a 5 s blotting using Vitrobot (ThermoFisher Scientific). After blotting, grids were rapidly immersed into pre-cooled liquid ethane and transferred to liquid nitrogen. Data acquisition was done on Titan Krios operating at 300 kV equipped with Gatan K3 Summit detector and GIF Quantum energy filter at 81,000× magnification. Movie stacks were automatically acquired using AutoEMation^52^ with a 20 eV slit width and a defocus range from −1.2 to −2.2 μm. Each stack consisting of 32 frames was exposed for 2.56 s with total dose of ~50 e^−^/Å^2^. The stacks were motion corrected with MotionCor2^53^ and binned two fold, resulting in a pixel size of 1.087 Å/pixel. The defocus values were estimated with Gctf^54^.

### Data processing

A diagram for data processing is presented in Fig. S1. A total of 10,530,363 particles were automatically picked by Gautomatch (developed by Kai Zhang, MRC-LMB) from 12,578 micrographs. Specifically, the particle picking procedure was carefully tuned and carried out in cycles using 2D averages from the current cycle as the picking template for the next cycle. To carefully tune the particle picking parameters including particle_diameter, cc_cutoff, local_average cutoff, local sigma cutoff and minimum inter particle distance, at least three representative micrographs were chosen for each defocus interval with a defocus value width of 1000 Å, and the parameters were manually adjusted until satisfactory results were obtained for the test data set. After particle extraction, three rounds of 2D classification gave rise to an initial data set with 9,504,206 particles. To achieve efficient and satisfactory initial model generation, this data set was then further reduced through 2D classification to obtain a data subset containing only 561,750 particles showing the best secondary structural features in the final results, and this data set was used in later procedures for generation of a good reference at 3.2 Å resolution.

The initial model was generated with cryoSparc^55^ and used as a reference for subsequent 3D classification. For initial model generation, the strong noise from micelle impeded proper alignment of the small membrane proteins to one single reference. To overcome this obstacle, we sought to minimize bias towards noise in the micelle region using an improved (https://github.com/hgxy15/relion-2.1_random_phase.git) phase-randomization strategy described previously^56^. In brief, the good reference was first masked with a tight protein mask to separate the protein signals and the micelle region. The micelle region was then subjected to phase randomization at each iteration and the signal within the protein mask was kept unchanged for the good reference. Conversely, the signal within the protein mask was subjected to phase randomization and the EM density in the micelle region was kept unchanged for the bad references. After pooling particles in the good class at each iteration, auto-refinement of the particles resulted in a reconstruction at 3.5 Å resolution using 413,506 particles after application of a protein mask that only included the transmembrane helices and extracellular regions.

We then used the 3.5 Å resolution reference to carry out several rounds of 3D classification on the 561,750 particles data set. During the “guided multi-reference classification” procedure, we used 3.5 Å resolution reconstruction as the best reference and some bad ones that were low-pass filtered or phase-randomized after subtraction of the protein signals. After pooling particles from the good class from the last several iterations. The steps above resulted in a map with more distinct features, with the final reconstruction arriving at an overall resolution at 3.2 Å using 121,490 particles (Supplementary Figs. 1, 2).

To achieve maximum utilization of the collected data set, the 3.2 Å reconstruction was then used as the good seed to find out the good particles within the original data set which contains 9,504,206 particles, specifically, several rounds of guided 3D classification was carried out in cryoSparc using the Heterogeneous Refinement job. Through pooling the good particles and further refinement using the Local-Refinement (New) job in cryoSparc, A final reconstruction at 2.2 Å was obtained from 8,482,332 particles (Supplementary Figs. 1, 2). The final reconstruction quality was further improved with CTF-refinement and Bayesian-polishing in relion-3.1^57,58^. The binning of the final reconstruction was updated to the super-resolution scale with a pixel size of 0.5435 Å during the Bayesian-polishing process. The resolution was estimated with the gold-standard Fourier shell correlation 0.143 criterion^59^ with high-resolution noise substitution^60^.

### Model building and refinement

The atomic coordinate of the WLS/Wnt3a complex was generated by combining homology modeling and de novo model building. An initial structure model of WLS TM2-8 and luminal domain was firstly predicted by trRosetta^32^ and tFold^33^. TM1 of WLS was generated using SWISS-MODEL online server^61^. The template used for the homology modeling of Wnt3a was hWnt3 from Wnt-frizzled (CRD) complex^42^ (PDB code: 6AHY). The model was manually adjusted in COOT^62^ according to the cryo-EM maps. Both 3.2 Å and 2.2 Å maps (Supplementary Fig. 1) were used for model building as EM density in some peripheral regions displayed better features in the 3.2 Å map compared to the final map at 2.2 Å resolution due to masking effect which arose from the mask used to improve map quality in the central core region.

The improved model of WLS/Wnt3a complex was refined against the corresponding map using PHENIX^63^ in real space with secondary structure and geometry restraints. Overfitting of model was monitored by refining the model in one of the two independent maps from the gold-standard refinement approach, and testing the refined model against the other map^64^.

### Mass spectrometry analysis

The SDS-PAGE was used to separate the protein and stained with Coomassie Blue G-250. The gel bands of interest were cut into pieces. Sample was digested by trypsin with prior reduction and alkylation in 50 mM ammonium bicarbonate at 37 °C overnight. The digested products were extracted twice with 1% formic acid in 50% acetonitrile aqueous solution and dried to reduce volume by speedvac.

For LC-MS/MS analysis, the peptides were separated by a 65 min gradient elution at a flow rate 0.300 µL/min with the Thermo EASY-nLC1200 integrated nano-HPLC system which is directly interfaced with the Thermo Q Exactive HF-X mass spectrometer. The analytical column was a home-made fused silica capillary column (75 µm ID, 150 mm length; Upchurch, Oak Harbor, WA) packed with C-18 resin (300 A, 3 µm, Varian, Lexington, MA). Mobile phase A consisted of 0.1% formic acid, and mobile phase B consisted of 100% acetonitrile and 0.1% formic acid. The mass spectrometer was operated in the data-dependent acquisition mode using the Xcalibur 4.1 software and there is a single full-scan mass spectrum in the Orbitrap (400–1800 m/z, 60,000 resolution) followed by 20 data-dependent MS/MS scans at 30% normalized collision energy. Each mass spectrum was analyzed using the Thermo Xcalibur Qual Browser and Proteome Discovery for the database searching.

### Immunoprecipitation

HeLa cells were seeded into 6-well plates the day before transfection. According to experimental purpose, BacMam-Wnt3A-10xhis and BacMam-WLS-Flag plasmids were co-transfected into Hela cells with different total amount and Wnt3a: WLS DNA ratio (total 1 µg at ratio 1: 1, total 2.5 µg at ratio 1: 1, or total 2.5 µg at ratio 4: 1). The ratio of DNA to Lipofectamine 3000 (Thermo Fisher) was 1: 2. 36 h after transfection, cells were havested and lysed with Lysis buffer (25 mM Tris-HCl pH 8.0, 150 mM NaCl, 1% Triton X-100, protease inhibitor cocktail) at 4 °C. Cell lysates were obtained by centrifugation at 15000 g for 10 mins. 3/4 of cell lysates were mixed with anti-Flag resin (GenScript). After 1.5 h of incubation, the beads were washed by wash buffer (25 mM Tris-HCl pH 8.0, 300 mM NaCl, 0.1% Triton X-100) for four times. Finally, the proteins on beads were analyzed by SDS-PAGE and western blotting. Experiments were repeated for at least three times. The intensities of blots were analyzed with ImageJ. Full scans of blots and raw data for blot intensities are included in the Source Data file. Information for antibodies is as follow: Rabbit polyclonal anti-Wnt3a antibody (Sigma-Aldrich, Cat. # 09-162, 1:1000 dilution); Rabbit polyclonal anti-WLS (Sangon Biotech, D264109, 1:1000 dilution); β-Actin (8H10D10) Mouse mAb (CST, Cat. #3700, 1:1000 dilution); Goat anti-Mouse IgG, HRP conjugated (CWBIO, CW0102, 1:10000 dilution); Goat-anti-Rabbit IgG, HRP conjugated (CWBIO, CW0103, 1:10000 dilution).

### Wnt/β-catenin TOPFlash assay

One day before transfection, Hela cells were seeded into 24-well plates. In total, 100 ng (or 300 ng) of BacMam-Wnt3a, 300 ng TOPFlash construct (Beyotime) and 6 ng Renilla luciferase expression construct were co-transfected into Hela cells with 1:2 ratio to lipofectamine 3000. After 36 h’ post-transfection, medium for different Wnt3a variants were collected for Wnt3A secretion detection by western blotting; cells were harvested, and luciferase activities were measured using Dual Luciferase Reporter Gene Assay Kit (Beyotime). Readings for Renilla luciferase activity were used as internal control for Wnt3a activity normalization. Experiments were repeated for at least three times. Full scans of blots and raw data for TOPFlash assay readings are included in the Source Data file.

### Reporting summary

Further information on research design is available in the Nature Research Reporting Summary linked to this article.

## Supplementary information

Supplementary Information

Reporting summary

## Data Availability

Data supporting the findings of this manuscript are available from the corresponding authors upon reasonable request. Atomic coordinate and corresponding EM maps of the WLS-Wnt3a complex PDB 7DRT and EMD-30827 have been deposited in the Protein Data Bank (http://www.rcsb.org) and the Electron Microscopy Data Bank (https://www.ebi.ac.uk/pdbe/emdb/), respectively. Source data are provided with this paper.

## Source data

Source Data
