# Supplementary material for: Cryo-EM structure of human Wntless in complex with Wnt3a
Source: Nat Commun. 2021 Jul 27;12:4541. doi: 10.1038/s41467-021-24731-3 (PMC8316347; doi:10.1038/s41467-021-24731-3)
Supplement: Supplementary file 1 — Supplementary Information [file 41467_2021_24731_MOESM1_ESM.pdf]

Supplementary Information for

Cryo-EM structure of human Wntless in complex with Wnt3a

Qing Zhong[#], Yanyu Zhao[#], Fangfei Ye[#], Zaiyu Xiao[#], Gaoxingyu Huang, Meng Xu, Yuanyuan Zhang, Ke Sun, Xiechao Zhan, Zhizhi Wang, Shanshan Cheng, Shan Feng, Xiuxiu Zhao, Jizhong Zhang, Peilong Lu, Wenqing Xu, Qiang Zhou*, Dan Ma*

∗To whom correspondence should be addressed: D. Ma (madan@westlake.edu.cn), Q. Zhou (zhouqiang@westlake.edu.cn)

This file includes:

Supplementary Figs. 1-11

Supplementary Tables 1-2

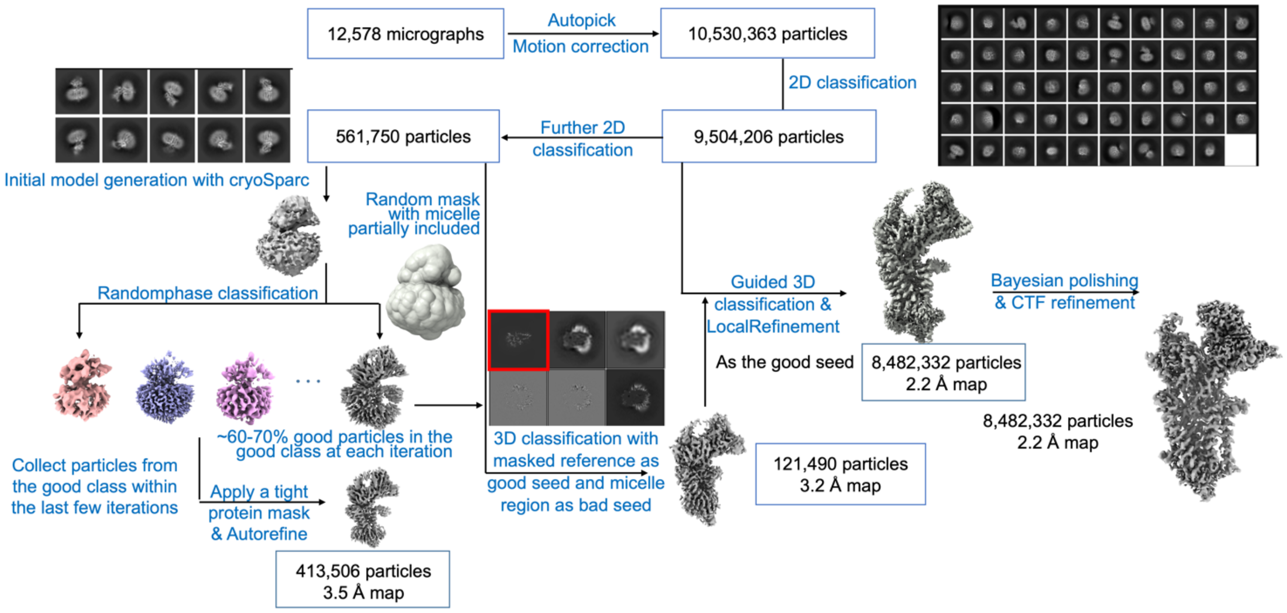

Supplementary Fig. 1 | Flow chart for cryo-EM analysis. For details, see 'Data Processing' section in Methods section.

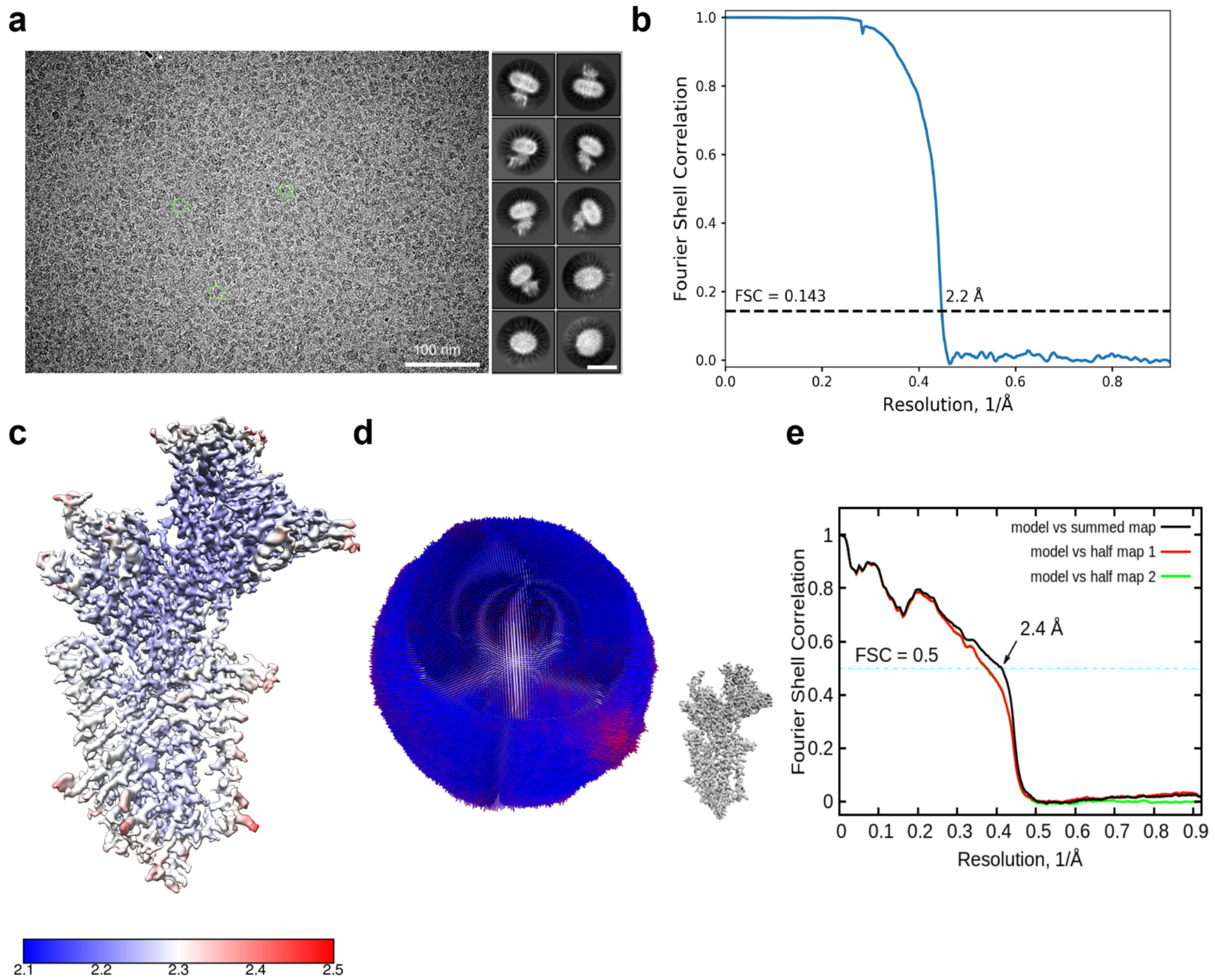

Supplementary Fig. 2 │ Cryo-EM data and map quality. a Representative cryo-EM micrograph and 2D class averages of particle images. The scale bar in 2D class averages is 10 nm. The data were independently collected for three times. **b** FSC curve of the Wnt3A-WLS complex. **c** Local resolution map. **d** Euler angle distribution in the final 3D reconstruction. **e** FSC curve between the map and the refined model.

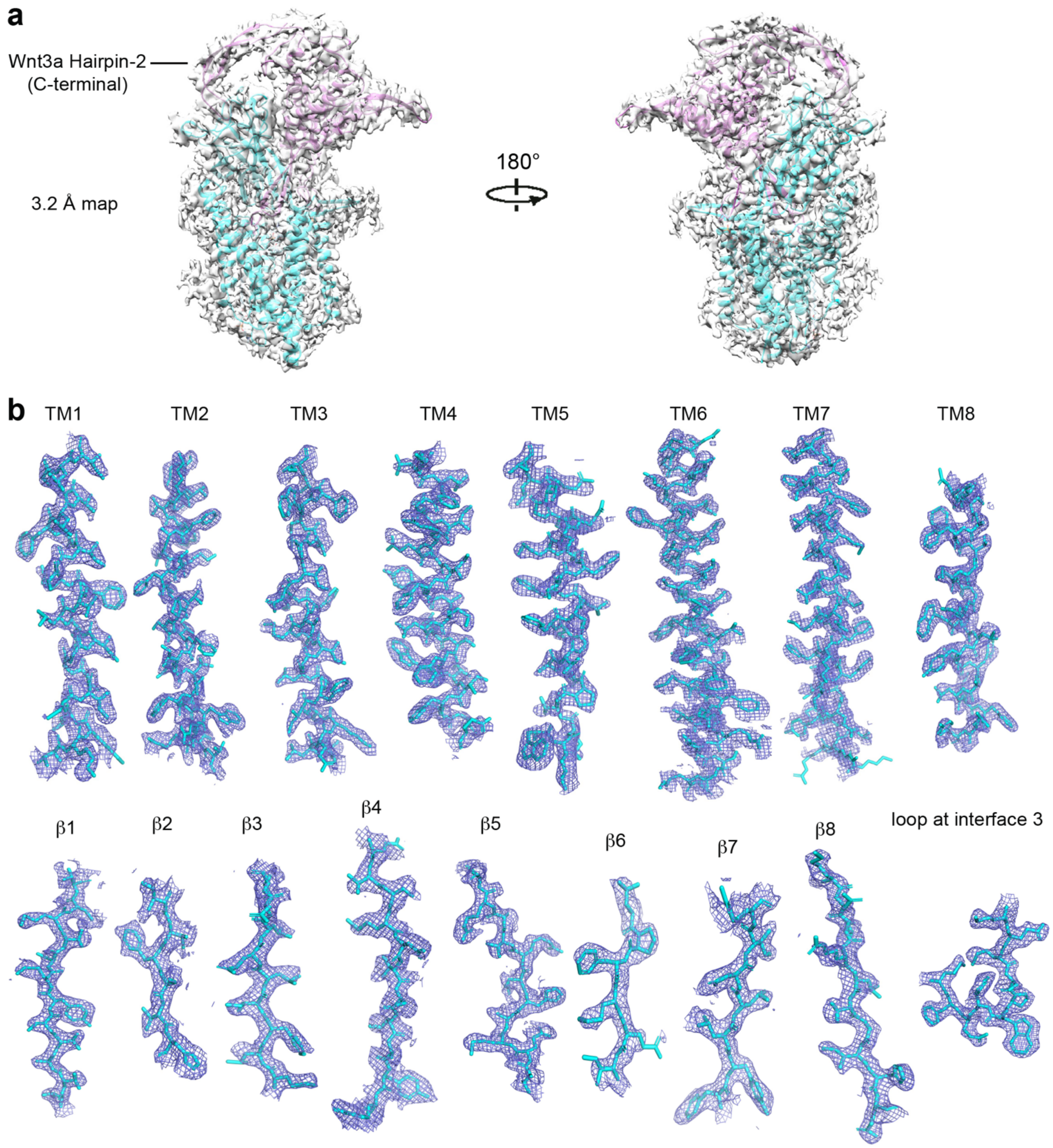

Supplementary Fig. 3 | Fit of complex and WLS model with cryo-EM density. a Fit of WLS-Wnt3a complex model with the 3.2 Å cryo-EM map. Wnt3a is colored in violet and WLS is colored in cyan. The map is generated from the 3.2 Å map in Chimera at contour level of 0.02 with dust hidden. **b** Cryo-EM densities (blue) are superimposed on transmembrane helices (TM) and loop at interface 3 of the WLS model with $\sigma = 7.0$, and beta strands (β) with $\sigma = 5.0$. The model is rendered in cyan sticks.

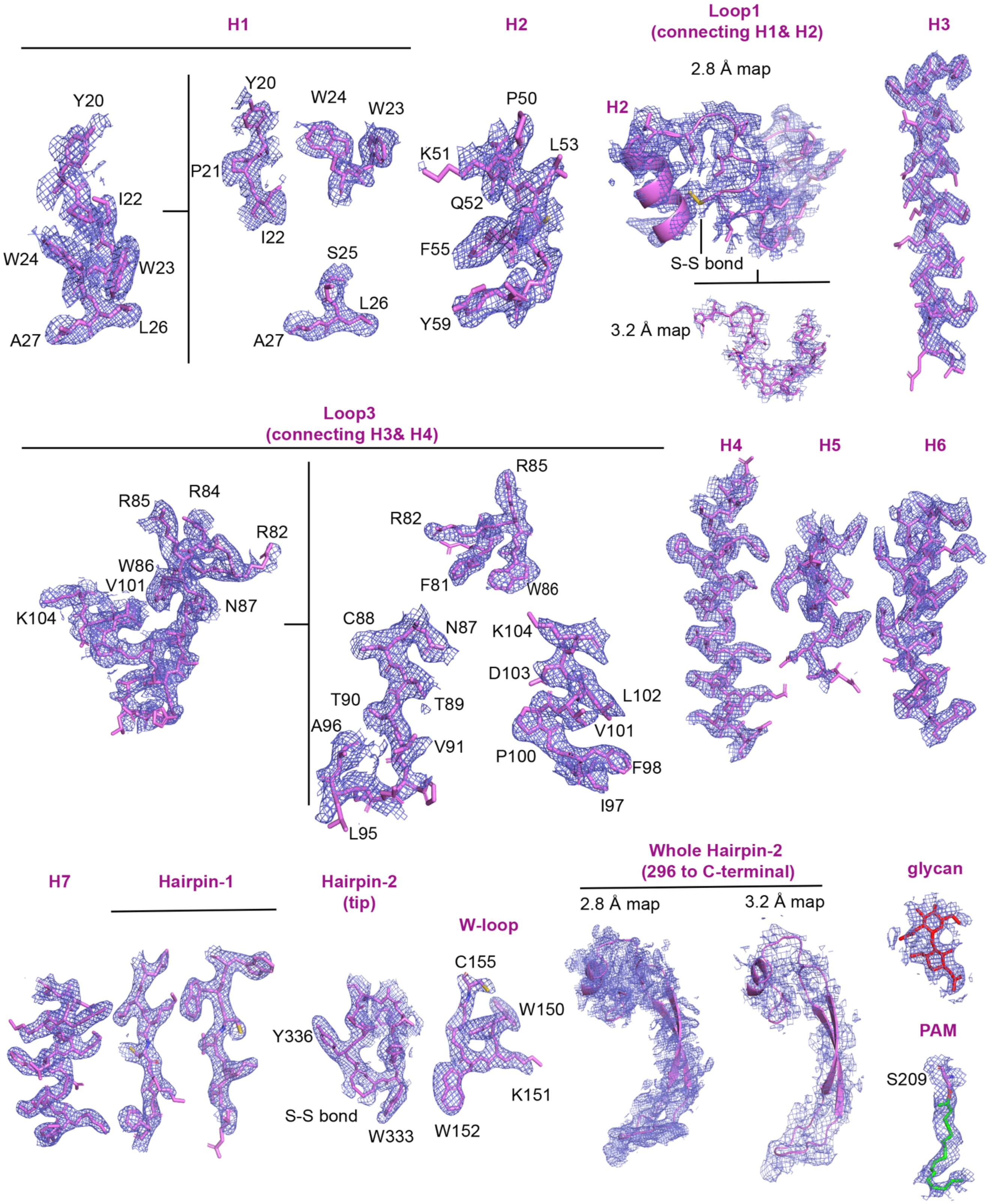

Supplementary Fig. 4 | Fit of Wnt3a model with cryo-EM density. The 2.2 Å map was low pass filtered into a 2.8 Å map for Wnt3a model fit map analysis. Cryo-EM densities (blue) are superimposed on helices (H), hairpins/loop at interfaces, Loop1, Loop3, glycan and PAM of the Wnt3a model. Both densities from the 2.8 Å map and 3.2 Å map are shown for Loop1 and whole Hairpin-2. Glycan and PAM are fitted into the 2.2 Å map. Figures are generated with PyMOL at σ = 2.0 for Loop1, whole Hairpin-2, glycan and PAM, σ = 5.0 for H1, H2, and Loop3, and σ = 10.0 for the rest. The model is rendered in violet sticks, with glycan in red and PAM in green.

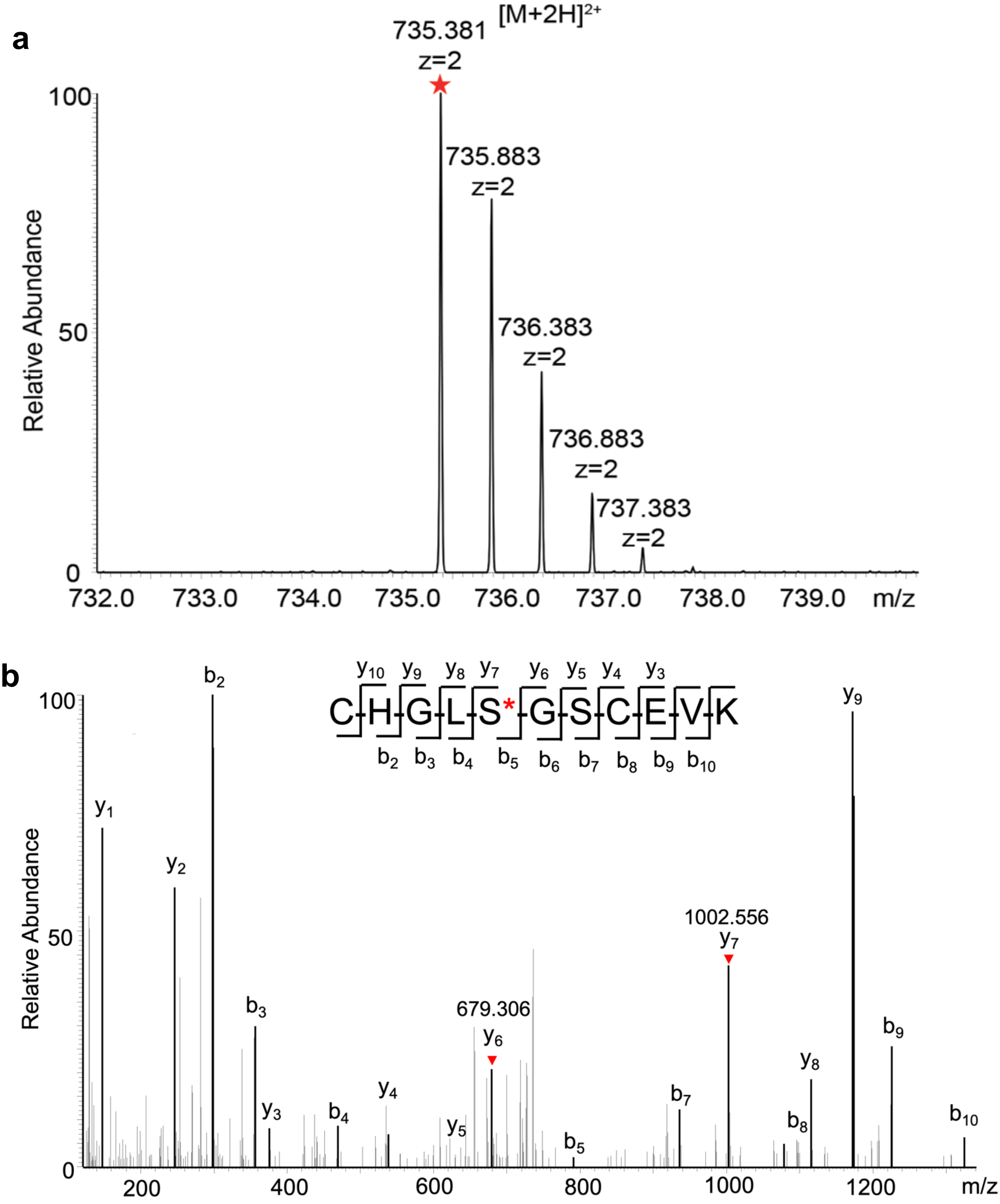

Supplementary Fig. 5 | Mass spectrometry validation of PAM modification on Wnt3a. a MS spectra of Wnt3a in cryo-EM sample at the m/z range from 732 to 739. The peak for tryptic peptide containing the PAM modified serine (sequence: CHGLSGSCEVK) is indicated with a star. **b** The MS/MS spectrum from the precursor ion at m/z 735.381 of Wnt3a. The value of y_7 minus y_6 equals to the molecular weight of palmitoleoylated serine.

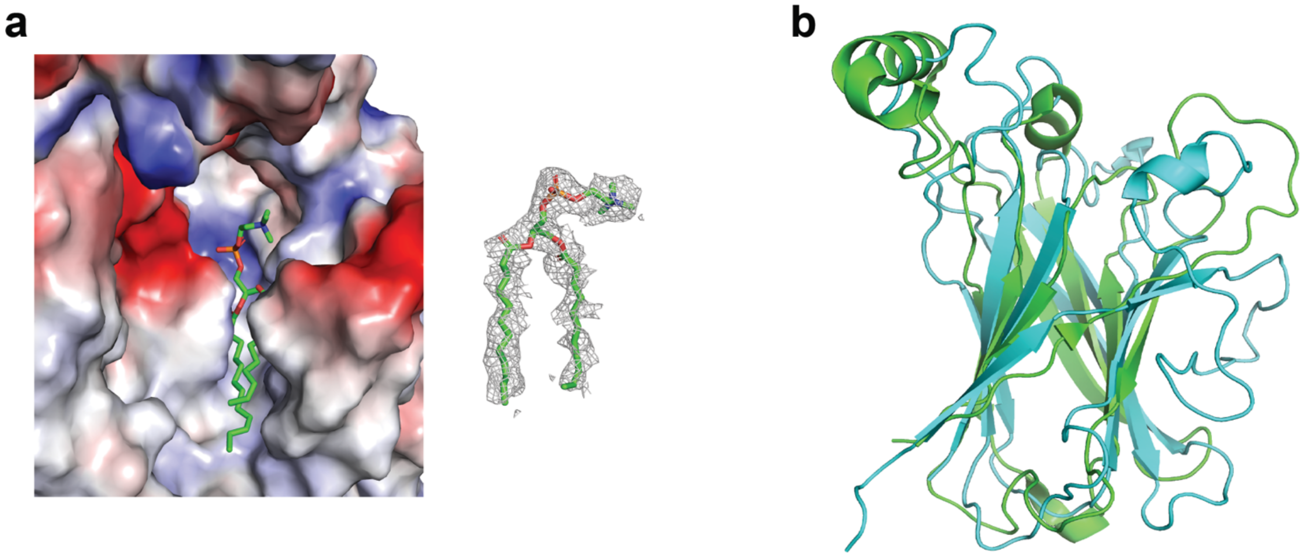

Supplementary Fig. 6 | WLS structural features. a A POPC molecule in the hydrophobic cavity of WLS TM domain. Structure model of the lipid fits well with cryo-EM density (σ = 2.0). **b** Structure superimposition of WLS luminal domain with Seipin. Result from Dali server. PDB code of Seipin structure: 6DS5. WLS luminal domain is colored in cyan and Seipin is colored in green.

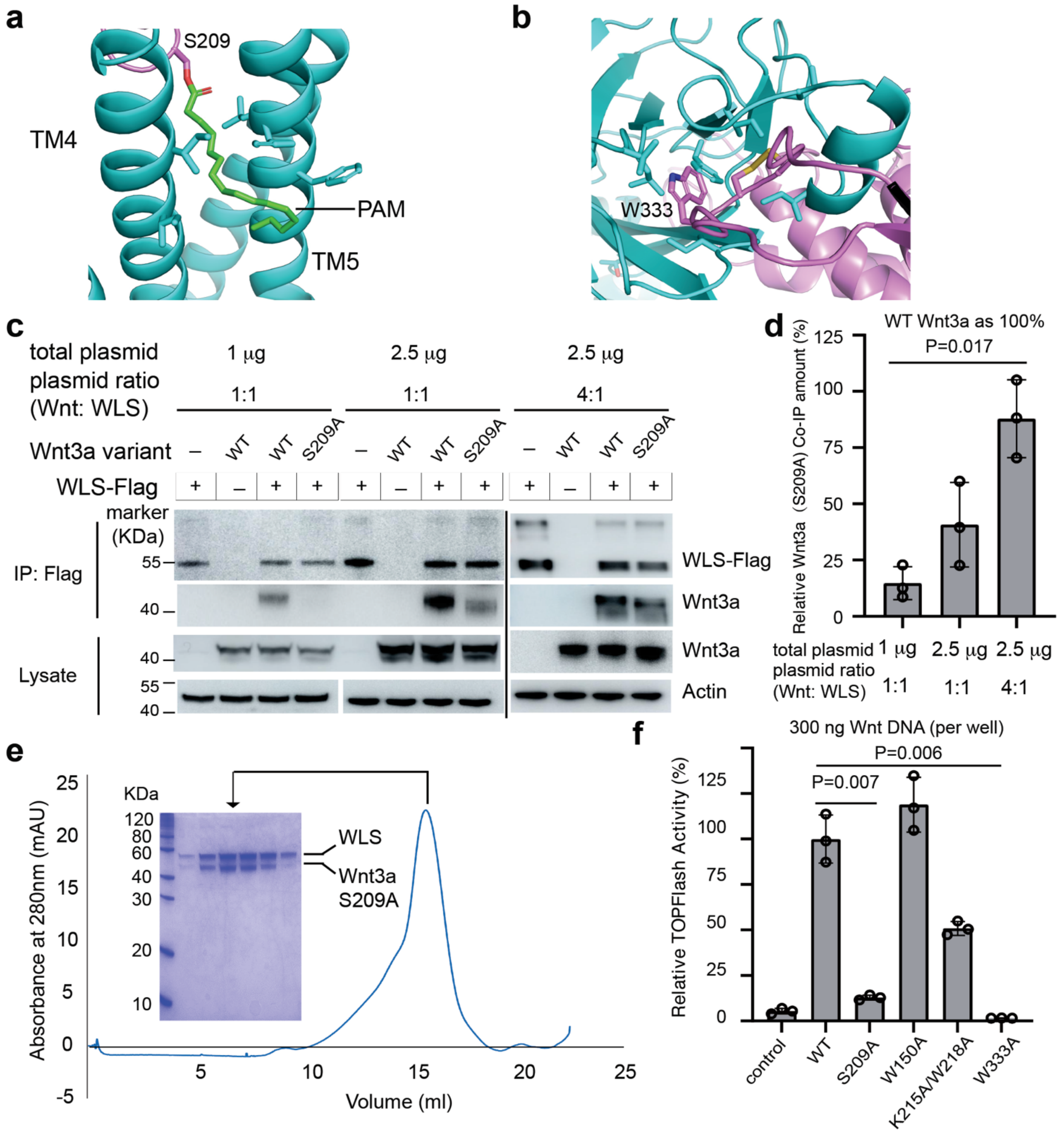

Supplementary Fig. 7 | Analysis of WLS association and signaling activity for Wnt3a mutants. a Interaction details between Wnt3a PAM and WLS. **b** Interaction details between Wnt3a Trp333 and WLS. **c** Co-IP analysis of Wnt3a S209A mutant binding with WLS. Co-IP of WT Wnt3a and Wnt3a S209A mutant with Flag-tagged WLS when different total amount of plasmids at different plasmid weight ratio were transfected into Hela cells. Experiments were independently performed for three times with similar results. **d** Percentage co-IP amount of Wnt3a S209A mutant compared to WT Wnt3a (100%). **e** Gel filtration profile (Superose 6 Increase 10/300 was used) and SDS-PAGE analysis of co-expressed/purified WLS-Wnt3a (S209A) complex. **f** Signaling activity measurement of Wnt3a mutants. Activity is shown as relative TOPFlash activity compared to WT Wnt3a when 300 ng Wnt3a DNA was transfected into cells per well (24-well plate). All histograms in panel **d** and **f** were generated from n=3 independent measurements by GraphPad Prism 9. Statistical analysis was performed by two-sided test; mean ± S.D.

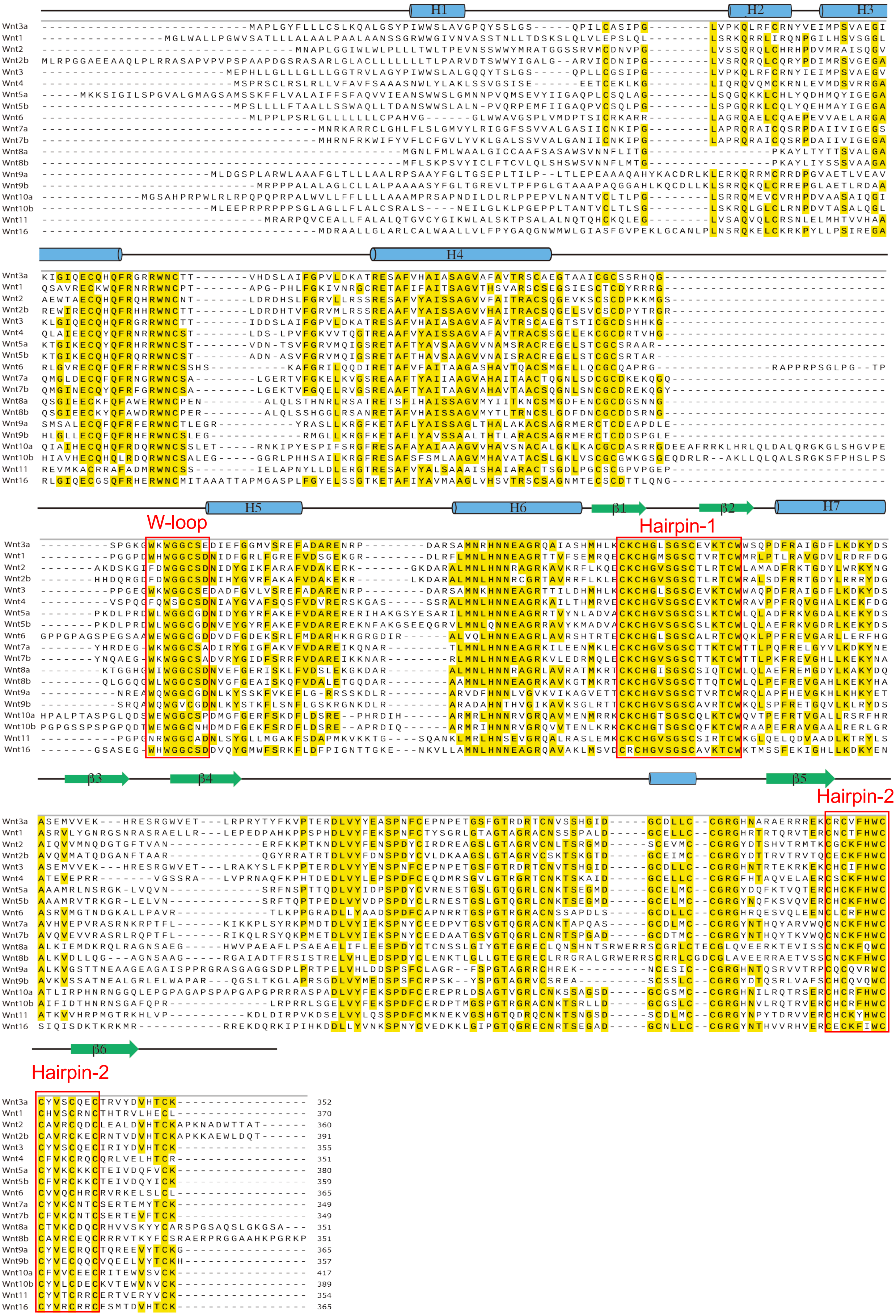

Supplementary Fig. 8 │ Sequence alignment of 19 human Wnts. Conserved residues are shaded in yellow. Hairpin-1, Hairpin-2 and W-loop are indicated with red boxes.

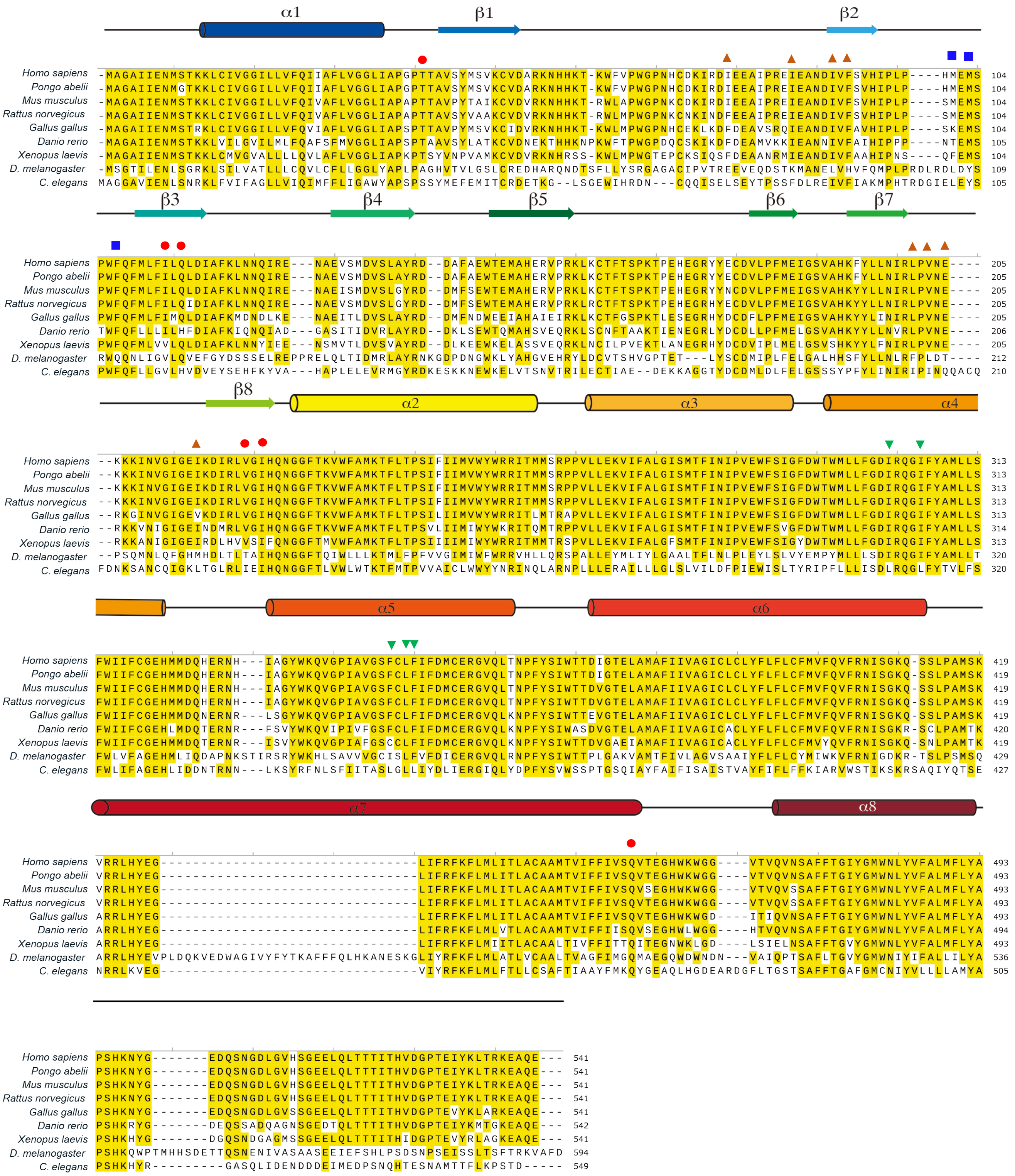

Supplementary Fig. 9 | Sequence alignment of WLS from 9 different species. Conserved residues are shaded in yellow. Major residues at interfaces between WLS and Wnt3a are highlighted with inverted green triangles for PAM interacting residues, red spots for rest interacting residues at interface 1, orange triangles for interface 2, and blue squares for interface 3, respectively.

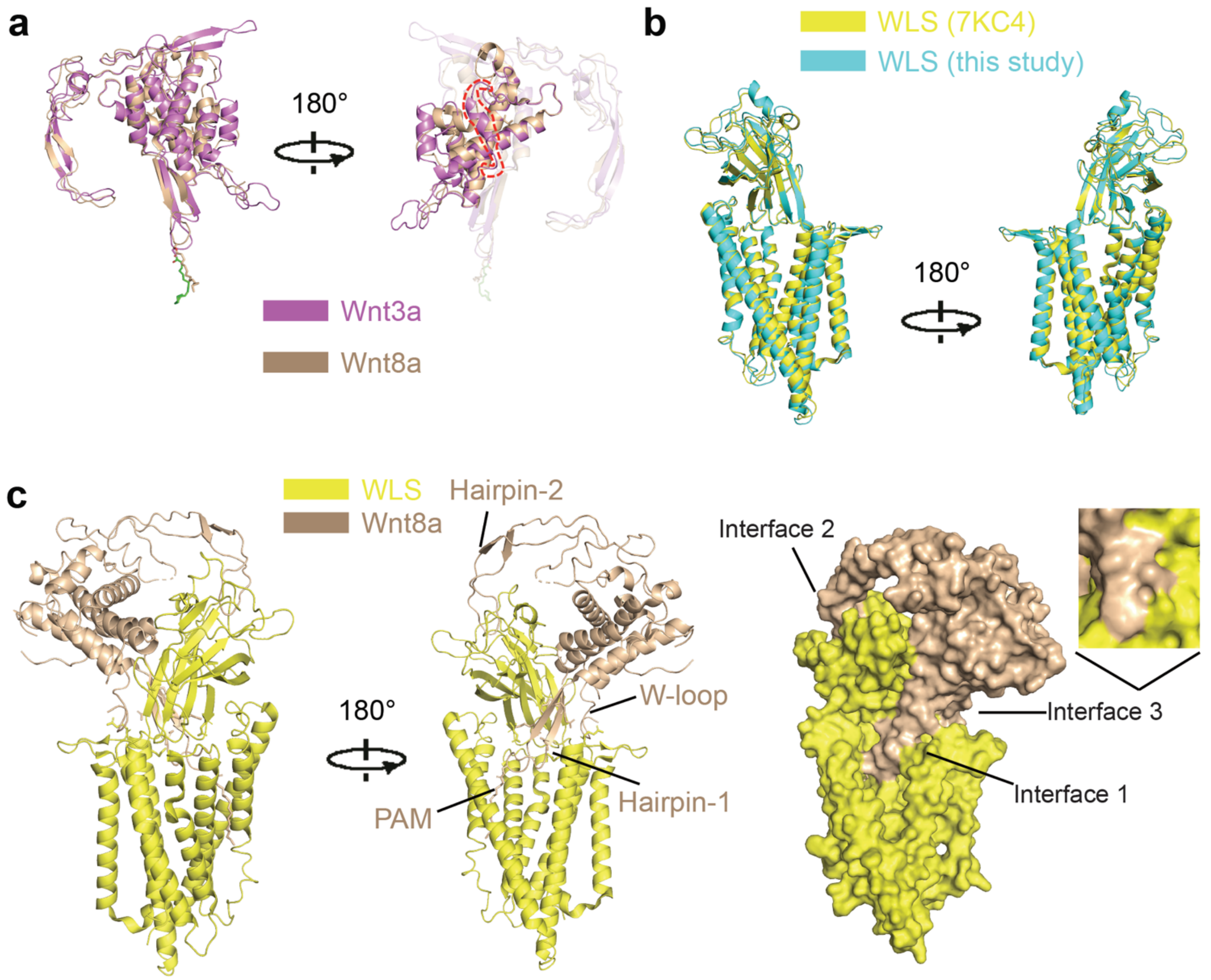

Supplementary Fig. 10 | Comparison of WLS-Wnt3a and WLS-Wnt8a structures. a Superimposition of Wnt3a (this study) and Wnt8a in 7KC4 (RMSD: 1.38 Å) with Wnt3a colored in violet and Wnt8a colored in wheat. In right panel, N-terminal regions are emphasized by adjusting transparency of remaining part as 80%. The extended N-terminal region in Wnt3a is highlighted by dashed red line. **b** Superimposition of Wnt3a-bound WLS (this study) and Wnt8a-bound WLS (RMSD: 1 Å). **c** WLS-Wnt8a share similar binding mode to WLS-Wnt3a. The three interfaces corresponding to that in WLS-Wnt3a are also mediated by corresponding Hairpin-1, Hairpin-2 and W-loop of Wnt8a in WLS-Wnt8a complex.

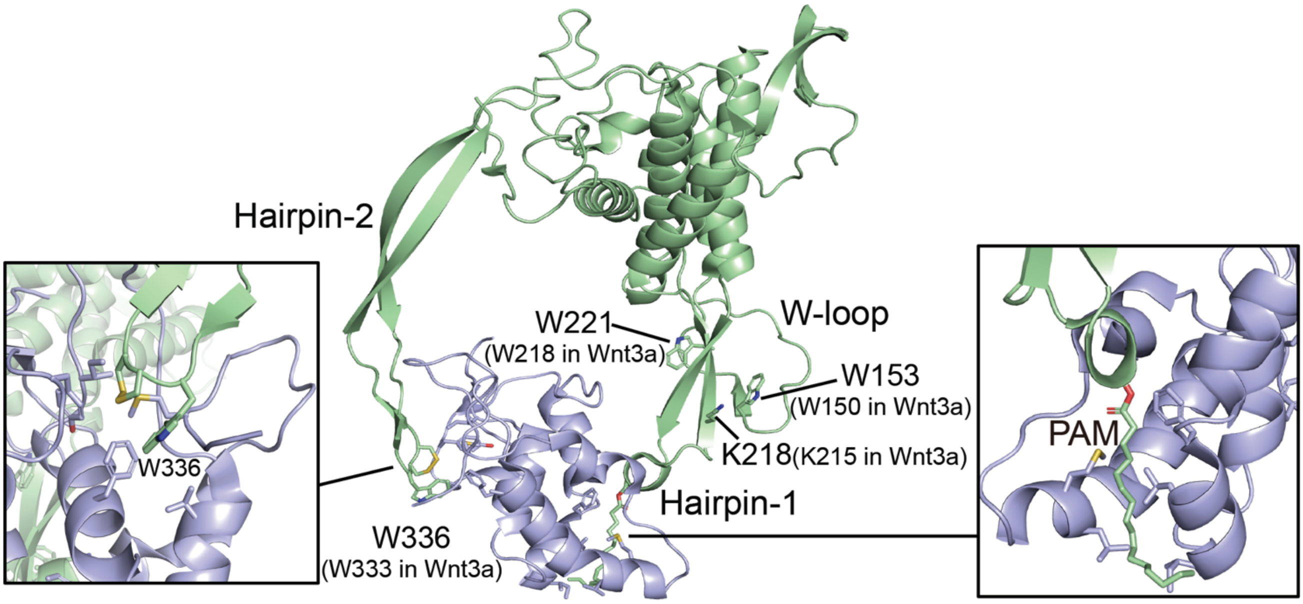

Supplementary Fig. 11 | Corresponding Wnt3a mutation sites in Wnt3-FZD structure. 6AHY structure model is used for the analysis with Wnt3 in pale green and FZD-CRD in purple.

Supplementary Table 1 | Cryo-EM data collection, refinement and validation statistics

**Data collection**	
EM equipment	Titan Krios (Thermo Fisher Scientific)
Voltage (kV)	300
Detector	Gatan K3 Summit
Energy filter	Gatan GIF Quantum, 20 eV slit
Pixel size (Å)	1.087 (0.5435 for super-resolution data)
Electron dose (e-/Å2)	50
Defocus range (μm)	-1.2 ~ -2.2
Data set	Wntless-Wnt3a complex
Number of images	12,578
**Reconstruction**	
Software	RELION 3.0
Initial number of particles	10530363
Number of used particles	8482332
Symmetry	C1
Final Resolution (Å)	2.2
Map resolution FSC threshold	0.143
Map sharpening B factor ($Å^2$)	-76.3
**Model building and refinement**	
Model building software	Coot
Refinement software	Rosetta / Phenix
Initial model	6AHY， predicted model from trRosetta, tFold and SWISS-MODEL
Model resolution FSC threshold	0.5
**Model composition**	
Protein residues	830
Wnt3a	334
Wntless	496
Side chains	770
Sugar	2
Lipid	5
**Validation**	
Clash score	4.99
Poor rotamers (%)	2.24
R.m.s deviations	
Bonds length (Å)	0.011
Bonds Angle (˚)	1.306
Ramachandran plot statistics (%)	
Preferred	94.19
Allowed	5.33
Outlier	0.48

Supplementary Table 2 | Primer information for cloning

construct	Forward (5'-3')	Reverse (5'-3')
Wnt3a-10xhis	tcaaaggcctacgtcgacATGGCCCCACTCGGATAC	caggctctagattcgaaaTTAATGGTGATGGTGATGG
Wnt3a-notag	tcaaaggcctacgtcgacATGGCCCCACTCGGATAC	caggctctagattcgaaaTTACTTGCATGTGTGCACGTC
Wnt3a-K215/W218A	GGCAGCTGCGAGGTGGCCACATGCGCCTGGTCGCAACCCGAC	GTCGGGTTGCGACCAGGCGCATGTGGCCACCTCGCAGCTGCC
Wnt3a-W333A	GCTGCGTGTTCCACGCCTGCTGCTACGTCAGCTG	GCTGACGTAGCAGCAGGCGTGGAACACGCAGC
Wnt3a-W150A	CTCACCAGGCAAGGGCGCCAAGTGGGGTGGCTGT	ACAGCCACCCCACTTGGCGCCCTTGCCTGGTGAG
WLS-Flag	tcaaaggcctacgtcgacATGGCTGGGGCAATTATAG	caggctctagattcgaaaTTACTTGTCATCGTCATC
Wnt3a-S209A	AAGTGCCACGGGCTGGCCGGCAGCTGCGAGGTG	CACCTCGCAGCTGCCGGCCAGCCCGTGGCACTT
PORCN-GFP-Flag	cggaattcaaaggccaccATGGCCACCTTTAGCCG	gagactgcaggctctagaTTATTTATCGTCATCGTC

Note: Sequences from plasmid vectors are shown in lowercase, and sequences from target genes are shown in uppercase.
