# Supplementary material for: Cryo-EM structure of human Wntless in complex with Wnt3a
Source: Nat Commun. 2021 Jul 27;12:4541. doi: 10.1038/s41467-021-24731-3 (PMC8316347; doi:10.1038/s41467-021-24731-3)
Supplement: Supplementary file 2 — Reporting summary [file 41467_2021_24731_MOESM2_ESM.pdf]

nature research

Corresponding author(s): Dan Ma, Qiang Zhou

Last updated by author(s): 2021-07-01

# Reporting Summary

Nature Research wishes to improve the reproducibility of the work that we publish. This form provides structure for consistency and transparency in reporting. For further information on Nature Research policies, see our Editorial Policies and the Editorial Policy Checklist.

Please do not complete any field with "not applicable" or n/a. Refer to the help text for what text to use if an item is not relevant to your study.
For final submission: please carefully check your responses for accuracy; you will not be able to make changes later.

## Statistics

For all statistical analyses, confirm that the following items are present in the figure legend, table legend, main text, or Methods section.

| n/a | Confirmed | |
|---|---|---|
| ☐ | ☒ | The exact sample size (*n*) for each experimental group/condition, given as a discrete number and unit of measurement |
| ☐ | ☒ | A statement on whether measurements were taken from distinct samples or whether the same sample was measured repeatedly |
| ☐ | ☒ | The statistical test(s) used AND whether they are one- or two-sided<br>*Only common tests should be described solely by name; describe more complex techniques in the Methods section.* |
| ☒ | ☐ | A description of all covariates tested |
| ☒ | ☐ | A description of any assumptions or corrections, such as tests of normality and adjustment for multiple comparisons |
| ☐ | ☒ | A full description of the statistical parameters including central tendency (e.g. means) or other basic estimates (e.g. regression coefficient) AND variation (e.g. standard deviation) or associated estimates of uncertainty (e.g. confidence intervals) |
| ☐ | ☒ | For null hypothesis testing, the test statistic (e.g. *F*, *t*, *r*) with confidence intervals, effect sizes, degrees of freedom and *P* value noted<br>*Give P values as exact values whenever suitable.* |
| ☒ | ☐ | For Bayesian analysis, information on the choice of priors and Markov chain Monte Carlo settings |
| ☒ | ☐ | For hierarchical and complex designs, identification of the appropriate level for tests and full reporting of outcomes |
| ☒ | ☐ | Estimates of effect sizes (e.g. Cohen's *d*, Pearson's *r*), indicating how they were calculated |

*Our web collection on statistics for biologists contains articles on many of the points above.*

## Software and code

Policy information about availability of computer code

| | |
|---|---|
| Data collection | AutoEMation for cryo-EM data collection, MotionCor 2 for motion correction, Gctf 1.06 for defocus estimation |
| Data analysis | Relion 3.1, PHENIX1.18.2, trRosetta, tFold, COOT, PyMOL 2.5, Chimera 1.15, ConSurf server, Dali server, SnapGene 5.2.3, GraphPad Prism 9, cryoSPARC 3.2, Gautomatch 0.56, ImageJ |

For manuscripts utilizing custom algorithms or software that are central to the research but not yet described in published literature, software must be made available to editors and reviewers. We strongly encourage code deposition in a community repository (e.g. GitHub). See the Nature Research guidelines for submitting code & software for further information.

## Data

Policy information about availability of data

All manuscripts must include a data availability statement. This statement should provide the following information, where applicable:
- Accession codes, unique identifiers, or web links for publicly available datasets
- A list of figures that have associated raw data
- A description of any restrictions on data availability

Data supporting the findings of this manuscript are available from the corresponding authors upon reasonable request. Atomic coordinate and corresponding EM maps of the WLS-Wnt3a complex PDB 7DRT (http://doi.org/10.2210/pdb7DRT/pdb) and EMD-30827 (https://www.ebi.ac.uk/pdbe/entry/emdb/EMD-30827) have been deposited in the Protein Data Bank (http://www.rcsb.org) and the Electron Microscopy Data Bank (https://www.ebi.ac.uk/pdbe/emdb/), respectively. Source data are provided with this paper.

# Field-specific reporting

Please select the one below that is the best fit for your research. If you are not sure, read the appropriate sections before making your selection.

- [x] Life sciences
- [ ] Behavioural & social sciences
- [ ] Ecological, evolutionary & environmental sciences

For a reference copy of the document with all sections, see nature.com/documents/nr-reporting-summary-flat.pdf

# Life sciences study design

All studies must disclose on these points even when the disclosure is negative.

| | |
|---|---|
| Sample size | All histograms were generated from three independent measurements. We did extensive individual preliminary tests to confirm the reproducibility under our final experimental conditions. |
| Data exclusions | No data exclusions. |
| Replication | Co-IP assay, Wnt secretion assay and TOPFlash assay were performed at least three times to confirm the results. Independent replication was done every two to three days. All attempts under our final experimental conditions were successful. |
| Randomization | N/A. We had enough control and the reproducibility was good. Our results were sufficient to demonstrate the accuracy. |
| Blinding | N/A. We had enough control and the reproducibility was good. Our results were sufficient to demonstrate the accuracy. |

# Reporting for specific materials, systems and methods

We require information from authors about some types of materials, experimental systems and methods used in many studies. Here, indicate whether each material, system or method listed is relevant to your study. If you are not sure if a list item applies to your research, read the appropriate section before selecting a response.

## Materials & experimental systems

| n/a | Involved in the study | |
|---|---|---|
| ☐ | ☒ | Antibodies |
| ☐ | ☒ | Eukaryotic cell lines |
| ☒ | ☐ | Palaeontology and archaeology |
| ☒ | ☐ | Animals and other organisms |
| ☒ | ☐ | Human research participants |
| ☒ | ☐ | Clinical data |
| ☒ | ☐ | Dual use research of concern |

## Methods

| n/a | Involved in the study | |
|---|---|---|
| ☒ | ☐ | ChIP-seq |
| ☒ | ☐ | Flow cytometry |
| ☒ | ☐ | MRI-based neuroimaging |

## Antibodies

| | |
|---|---|
| Antibodies used | Rabbit polyclonal anti-Wnt3a antibody (Sigma-Aldrich, Cat. # 09-162, 1:1000 dilution); Rabbit polyclonal anti-WLS (Sangon Biotech, D264109, 1:1000 dilution); beta-Actin (8H10D10) Mouse mAb (CST, Cat. #3700, 1:1000 dilution); Goat anti-Mouse IgG, HRP conjugated (CWBIO, CW0102, 1:10000 dilution); Goat-anti-Rabbit IgG, HRP conjugated (CWBIO, CW0103, 1:10000 dilution). |
| Validation | All primary antibodies target human proteins in this study. We validated these antibodies by western blotting through obtaining bands with expected molecular weight. |

## Eukaryotic cell lines

Policy information about cell lines

| | |
|---|---|
| Cell line source(s) | HEK293S GnTI$^-$ and HeLa cell lines were from ATCC. |
| Authentication | No further authentication was performed for commercially available cell lines. |
| Mycoplasma contamination | No mycoplasma contamination. |
| Commonly misidentified lines (See ICLAC register) | No commonly misidentified cell lines were used. |

This checklist template is licensed under a Creative Commons Attribution 4.0 International License, which permits use, sharing, adaptation, distribution and reproduction in any medium or format, as long as you give appropriate credit to the original author(s) and the source, provide a link to the Creative Commons license, and indicate if changes were made. The images or other third party material in this article are included in the article's Creative Commons license, unless indicated otherwise in a credit line to the material. If material is not included in the article's Creative Commons license and your intended use is not permitted by statutory regulation or exceeds the permitted use, you will need to obtain permission directly from the copyright holder. To view a copy of this license, visit http://creativecommons.org/licenses/by/4.0/
